# Using a Hybrid of AI and Template-Based Method in Automatic Item Generation to Create Multiple-Choice Questions in Medical Education: Hybrid AIG

**DOI:** 10.2196/65726

**Published:** 2025-04-04

**Authors:** Yavuz Selim Kıyak, Andrzej A Kononowicz

**Affiliations:** 1 Department of Medical Education and Informatics, Faculty of Medicine, Gazi University, Ankara, Turkey; 2 Department of Bioinformatics and Telemedicine, Jagiellonian University Medical College, Medyczna Str 7, Kraków, 30-688, Poland, 48 12 3476908

**Keywords:** automatic item generation, ChatGPT, artificial intelligence, large language models, medical education, AI, hybrid, template-based method, hybrid AIG, mixed-method, multiple-choice question, multiple-choice, human-AI collaboration, human-AI, medical education, algorithm, expert

## Abstract

**Background:**

Template-based automatic item generation (AIG) is more efficient than traditional item writing but it still heavily relies on expert effort in model development. While nontemplate-based AIG, leveraging artificial intelligence (AI), offers efficiency, it faces accuracy challenges. Medical education, a field that relies heavily on both formative and summative assessments with multiple choice questions, is in dire need of AI-based support for the efficient automatic generation of items.

**Objective:**

We aimed to propose a hybrid AIG to demonstrate whether it is possible to generate item templates using AI in the field of medical education.

**Methods:**

This is a mixed-methods methodological study with proof-of-concept elements. We propose the hybrid AIG method as a structured series of interactions between a human subject matter expert and AI, designed as a collaborative authoring effort. The method leverages AI to generate item models (templates) and cognitive models to combine the advantages of the two AIG approaches. To demonstrate how to create item models using hybrid AIG, we used 2 medical multiple-choice questions: one on respiratory infections in adults and another on acute allergic reactions in the pediatric population.

**Results:**

The hybrid AIG method we propose consists of 7 steps. The first 5 steps are performed by an expert in a customized AI environment. These involve providing a parent item, identifying elements for manipulation, selecting options and assigning values to elements, and generating the cognitive model. After a final expert review (Step 6), the content in the template can be used for item generation through a traditional (non-AI) software (Step 7). We showed that AI is capable of generating item templates for AIG under the control of a human expert in only 10 minutes. Leveraging AI in template development made it less challenging.

**Conclusions:**

The hybrid AIG method transcends the traditional template-based approach by marrying the “art” that comes from AI as a “black box” with the “science” of algorithmic generation under the oversight of expert as a “marriage registrar”. It does not only capitalize on the strengths of both approaches but also mitigates their weaknesses, offering a human-AI collaboration to increase efficiency in medical education.

## Introduction

Multiple-choice items are integral to written assessment in medical education, serving as a useful tool for assessing a wide range of knowledge and skills. Their common use spans from evaluating factual knowledge to clinical reasoning and decision-making in various domains [1]. This assessment format supports high-volume testing with the added advantage of automated scoring to enhance the efficiency of examinations in medical education.

The traditional way of writing multiple-choice items, characterized by manual development processes, presents significant challenges in scalability. This challenge stems from the intensive time and effort required to create and review each question. This laborious process, which demands expertise and resources, faces a bottleneck in scaling up to meet the demand for a vast quantity of quality items. The challenge is particularly pronounced in medical education, where only a progress test administration in a year requires having 2400 multiple-choice items [2], showing the inefficiency of traditional methods in satisfying the needs of question banks in medical schools.

Template-based automatic item generation (AIG) is a scalable method used in educational assessment that leverages predefined templates to systematically produce multiple-choice items with the help of software [3]. It has been implemented in 7 languages [3-6] and different health professions [3,7-9]. It consists of 3 sequential stages: development of a cognitive model, development of an item model (template), and using software for the rapid generation of hundreds of items [10]. Item models developed based on cognitive models are structured by subject matter experts to include variables and rules for item generation, allowing for a more efficient creation of consistent questions. This approach enhances the scalability of item development and review compared with traditional item writing [3], addressing the demand for high-quality assessment materials across various educational domains. Template-based AIG, while generating high-quality multiple-choice items efficiently [11-15], still heavily relies on expert effort in development of cognitive models and item models. Although it depends on the content area and the expert’s skills, a study reported that development of a cognitive model and an item model can take hours for a subject matter expert [10]. Furthermore, this development process necessitates high levels of extraneous cognitive load due to high element interactivity [16], which requires experts to deal with several components simultaneously. Therefore, “creating cognitive models for AIG is challenging” [3] and it “requires a lot of practice” for experts [3].

On the other hand, nontemplate-based AIG, which “can be guided by the syntactic, semantic, or sequential structure of a text” [3], is an approach that leverages natural language processing to generate assessment items without relying on predefined templates. Unlike the template-based method, this method uses the ability of artificial intelligence (AI) to generate content dynamically, for example, using ChatGPT, which is an AI-based chatbot developed by OpenAI, for creating items based on specific topics or learning outcomes provided by users [17-21]. This approach allows for the generation of diverse and complex questions in seconds, offering flexibility and efficiency in item development. However, this AI-driven approach struggles with issues of inaccuracy and inconsistency [18], especially when good prompting strategies [22] are not used [23]. In AI-driven item generation, such as with ChatGPT, these issues often emerge due to the model’s reliance on its training data, which may not always align perfectly with the specific objectives intended by educators. For example, an AI might generate content that includes incorrect information, such as asserting that “the human heart only has two chambers” [24], or misinterpret the complexity level required for a medical education context. Furthermore, the “black box” [25] nature of these AI models complicates diagnosing and correcting these errors within the AI mechanism, as it is challenging to trace back how the AI arrived at a particular output. Therefore, it raises concerns regarding validity, reliability, transparency, fairness, and equity [26], and the process still requires subject matter experts to review and revise each generated question [18,19,21,22,27]. Although it is more efficient than traditional item writing, necessity for reviewing each question is still inefficient.

As outlined above, recent advancements in AIG have offered efficiency, yet each method—template-based and nontemplate-based—brings its own set of limitations. The gap, therefore, lies in the need for a method that merges the structured efficiency of template-based AIG with the content generation capabilities of AI-driven, nontemplate-based, approaches. This convergence could potentially address the pressing need for tools augmenting capabilities of medical educators in test development. We are aware of the interdependence of social (human) and technical elements within an organization [28], advocating for the design of systems that concurrently optimize both human and technological components to achieve effective outcomes. In AIG, this can be interpreted as the need to harmonize the collaboration of a subject matter expert and AI tools working together on developing item and cognitive models.

In this paper, we propose a hybrid AIG method that uses AI to generate an item model (template) and a cognitive model for applying the item template in a template-based item generation process. This capitalizes on the strengths of both approaches but also mitigates their respective weaknesses, offering a novel human-AI collaboration to increase AIG efficiency in medical education.

## Methods

### Study Process

This is a proof-of-concept study. Drawing on existing guidance on prompt engineering [29,30] and our experience in building human-AI dialogues [20,31], we developed a series of flipped interactions through a series of iterative refinements. In this approach, the AI initiates the conversation and prompts the human expert to provide the necessary data, enabling a collaborative process for generating and refining item templates. The AI shoulders the significant cognitive load involved in template development, thereby reducing the cognitive burden on experts, allowing them to focus on deciding which elements of the question are essential for assessing students’ knowledge.

We used GPT Builder (OpenAI), a platform for customizing ChatGPT to the user’s needs [32], to train our Custom GPT. Since we conducted this study in February 2024, the Custom GPT worked based on the frontier model at that time, GPT-4.

We will present our approach in a manner analogous to how Gierl et al [10] described the template-based AIG. To illustrate the process, we used two items: one from Gierl et al’s work [3], and another from a multiple-choice question (MCQ) similar to an item in the Turkish National Medical Specialty Exam, TUS (2021/1, clinical question number 58). The reason for choosing Gierl et al’s [3] item is that it allows readers to compare it with the existing item model developed by a subject matter expert. The reason for choosing a TUS item is that Gierl et al’s [3] item model has likely already been processed by ChatGPT, so we also aimed to focus on an item that had not been modelled before.

### Ethical Considerations

This study did not involve human subjects, patient data, or personal identifiers, and therefore did not require ethical approval. No informed consent was necessary as no human participants were involved. The study is based on the demonstration of a methodological approach using AI for automatic item generation.

## Results

### Hybrid AIG and Prompts

The Hybrid AIG consists of 7 steps, with the last 2 steps carried out outside the AI environment. The AI environment requires a specialized GPT trained for generating item models and cognitive models. The Custom GPT we developed is titled “Item Model Maker for AIG” and is accessible at [33]..

In addition, the prompt we used is provided in Multimedia Appendix 1.

### Step 1: Providing a Parent Item

The starting point for developing both the item model and the cognitive model in the hybrid AIG is a parent item, reflecting the approach used in the template-based method. It functions as a prototype for generating new questions that follow the template’s structure.

In the first step of the hybrid AIG, the expert provides a parent item to AI. Although it is not a requirement, it would be better if the parent item has been chosen from well-performing items in an exam (eg, a published item from a past medical licensure examination). This could mitigate the possible problems that can stem from the lack of quality in the parent item. If an expert does not have a parent item, they can use AI tools, such as Case-Based MCQ Generator [20], to generate an MCQ as a parent item.

The purpose of generating MCQs in medical education is for a human expert to assess the students’ skills and knowledge and to evaluate whether they have grasped the nuances of the information presented, aiming for outcomes that are consistent with expert reasoning. The parent item includes the necessary components and embedded possible cognitive models required for this purpose, as it is approved and provided by the expert. It serves as the well-established basis and acts as a seed for generating a new template. In the subsequent steps, AI will primarily handle the cognitive work in using one of the possible underlying cognitive models in the parent item to generate a template. The expert’s role will be to monitor and ensure the AI maintains a consistent cognitive model in its output.

We present below the parent items provided to AI, adopting them from the sources described in the Methods section.

Gierl et al’s [3] item:

“A 22-year-old female sees her doctor and reports that she’s been experiencing a mild cough and slight body aches that have developed over a few days.

Upon examination, she presents with an oral temperature of 37 °C . What is the most likely diagnosis?

- Hay fever
- Ear infection
- Common cold
- Acute sinusitis
- Seasonal influenza ”

TUS Item:

“A fifteen-year-old girl, who became ill during her physical education class after lunch, is brought to the infirmary complaining of coughing, shortness of breath, dizziness, facial swelling and redness, as well as itching in her hands and feet. During the physical examination, the patient appears anxious, with a blood pressure reading of 80/50 mmHg, periorbital edema, and wheezing upon auscultation. Which of the following is the most likely diagnosis for this patient?

- Exercise-induced asthma
- Cholinergic urticaria
- Pulmonary embolism
- Anaphylaxis
- Vasovagal syncope ”

### Step 2: Identification of Elements for Manipulation

In the next step, the expert identifies words or phrases in the parent item for variable manipulation.

In the identification process, the expert can, preferably, consider the importance of the content in the case that is more influential in deciding what the correct answer is. Table 1 shows AI’s response and what the expert chose as the elements for manipulation.

**Table 1.** Identification of elements for manipulation.

Who speaks	Gierl et al’s [3] item	TUS item
AI^a^	Thank you for sharing the parent item. Could you please identify words or phrases in the question that you would like to manipulate for variable creation? These can include demographic details, symptoms, examination findings, or any other elements that could vary across different versions of this question.	Based on the parent item you’ve provided, let’s proceed to the next step. Could you please identify words or phrases in the scenario and question that you would like to have as variable elements for manipulation? These could be specific symptoms, the patient’s demographics, the setting, or any other details you think should be variable.
Expert	Element 1: mild cough Element 2: slight body aches Element 3: over a few days Element 4: 37°C	Element 1: during her physical education class after lunch Element 2: coughing, shortness of breath, dizziness, facial swelling and redness Element 3: a blood pressure reading of 80/50 mm Hg, periorbital edema, and wheezing upon auscultation

a AI: artificial intelligence.

### Step 3: Replacement of Elements and Choosing Options

In this step, AI replaces the words and phrases received from the expert with the elements in brackets, then the expert reviews the output, and prompts AI to make necessary changes, if any. Subsequently, the expert chooses 2 or more options from the options in the parent item. The AI will use them to define the possible values of elements. Table 2 shows the replaced version and the expert’s choice among the options.

**Table 2.** Replacement of elements and choosing options.

Who speaks	Gierl et al’s [3] item	TUS item
AI^a^	Based on the elements you’ve identified, here’s how the parent item could be modified with these variables in brackets: "A 22-year-old female sees her doctor and reports that she’s been experiencing a [Element 1] and [Element 2] that have developed [Element 3]. Upon examination, she presents with an oral temperature of [Element 4]. What is the most likely diagnosis? A. Hay fever B. Ear infection C. Common cold D. Acute sinusitis E. Seasonal influenza” Could you please choose two or more options from the parent item that the variables will be focused on? This will help in offering variables for each element that significantly impact the correct answer according to medical sources and guidelines.	Great, I’ll incorporate these elements as variables into the parent item. Here’s how it will look: "A fifteen-year-old girl, who became ill [Element 1], is brought to the infirmary complaining of [Element 2], as well as itching in her hands and feet. During the physical examination, the patient appears anxious, with [Element3]. Which of the following is the most likely diagnosis for this patient? A. Exercise-induced asthma B. Cholinergic urticaria C. Pulmonary embolism D. Anaphylaxis E. Vasovagal syncope” Now, could you please choose two or more options from the answer choices that you’d like the variables to significantly impact? This will help in designing the variables for each element to ensure they align closely with the medical scenarios corresponding to the chosen options.
Expert	Common cold Seasonal influenza	Cholinergic urticaria Anaphylaxis

a AI: artificial intelligence.

### Step 4: Offering Values in Elements

At this stage, AI suggest possible values for each element that can impact the correct answer. Then, the expert reviews the output and makes necessary changes, if any, by providing prompts. Table 3 shows the response of AI in order for offering values. It is important to note that “values” were presented as “variables” by AI in the example because it did not work well when we call them “values” (eg, it was prone to offer numbers rather than words). It may not be a problem in newer versions.

**Table 3.** Artificial intelligence’s (AI’s) offer for values.

Who speaks	Gierl et al’s [3] item	TUS item
AI	Thank you for choosing “Common cold” and “Seasonal influenza” as the focus. Based on these options, I’ll offer variables for each element that significantly impact the correct answer, considering medical sources and guidelines: Element 1: Cough Variable 1: Persistent dry cough Variable 2: Mild cough Variable 3: Severe hacking cough Element 2: Body aches Variable 1: Slight body aches Variable 2: Moderate body aches Variable 3: Severe muscle pains Element 3: Duration of symptoms Variable 1: Suddenly over a few hours Variable 2: Over a few days Variable 3: Gradually over more than a week Element 4: Oral temperature Variable 1: 37°C (normal) Variable 2: 37.5°C to 38°C (mild fever) Variable 3: Over 38°C (fever) Do these variables seem appropriate for creating different versions of the MCQ^a^ item focused on distinguishing between the common cold and seasonal influenza? If you need any adjustments or have additional suggestions, please let me know!	Thank you for choosing options B (Cholinergic urticaria) and D (Anaphylaxis). Based on these selections, I’ll offer variables for each element that significantly impact the correct answer considering the chosen options. Here are the proposed variables: Element 1: Timing of symptoms During her physical education class after lunch. Immediately after taking a hot shower. While sitting in a hot classroom. After exposure to cold wind post-exercise. During a stressful exam. Element 2: Symptoms Coughing, shortness of breath, dizziness, facial swelling and redness. Generalized hives, sweating, and palpitations Localized itching and redness following sweating. Throat tightness, difficulty swallowing, and a sense of impending doom. Flushing, headache, and nausea after eating specific foods. Element 3: Clinical findings A blood pressure reading of 80/50 mm Hg, periorbital edema, and wheezing upon auscultation. Tachycardia with a blood pressure reading of 100/70 mmHg, and diffuse hives. Normal blood pressure, with localized swelling and erythema. Rapid drop in blood pressure upon exposure to an allergen, with angioedema. Elevated blood pressure due to anxiety, with mild facial edema. Please review these variables and let me know if they are appropriate or if you would like any modifications.

a MCQ: multiple-choice question.

### Step 5: Generating the Cognitive Model

In the following step, AI determines the constraints based on values (called “variables” by AI) approved by the expert, in order to create the cognitive model. Then, the expert reviews the output and prompts AI for necessary changes, if any. The presence of the expert is to validate the output, as in the previous steps.

Compared with the cognitive model in the traditional template-based method, our hybrid method adopts a simpler approach. By providing constraints, it allows to determine what items will be generated and what the correct option is in each item. Tables4  5 present the cognitive models. Although the content needs small adjustments that can be easily carried out by the expert (eg, the expert may ask ChatGPT to remove “37.5°C to 38 °C” to make the correct answers more evident), we did not make any changes to present ChatGPT’s original output in order for demonstrating that it is able to provide a useful cognitive model to begin with for further revisions. It is also possible due to the nature of GPT models that the output might defer in formatting as visible in the tables.

**Table 4.** Cognitive model generated by artificial intelligence (AI) for Gierl et al.’s [3] item.

Element (variable)	Constraints (supported options)
Cough	Persistent dry cough (seasonal Influenza) Mild cough (common cold) Severe hacking cough (seasonal influenza)
Body aches	Slight body aches (common cold) Moderate body aches (seasonal influenza) Severe muscle pains (seasonal influenza)
Duration of symptoms	Suddenly over a few hours (seasonal influenza) Over a few days (common cold) Gradually over more than a week (common cold)
Oral temperature	37°C (normal) (common cold) 37.5°C to 38°C (mild fever) (seasonal influenza) Over 38°C (fever) (seasonal influenza)

**Table 5.** Cognitive model generated by artificial intelligence (AI) for TUS item.

Variable element	Constraints (supported options)
Timing of symptoms	B and D
During her physical education class after lunch.	D
Immediately after taking a hot shower.	B
While sitting in a hot classroom.	B
After exposure to cold wind postexercise.	D
During a stressful exam.	B
Symptoms	B and D
Coughing, shortness of breath, dizziness, and facial swelling and redness.	D
Generalized hives, sweating, and palpitations.	B and D
Localized itching and redness following sweating.	B
Throat tightness, difficulty swallowing, and a sense of impending doom.	D
Flushing, headache, and nausea after eating specific foods.	D
Clinical findings	B and D
A blood pressure reading of 80/50 mmHg, periorbital edema, and wheezing upon auscultation.	D
Tachycardia with a blood pressure reading of 100/70 mmHg, and diffuse hives.	B and D
Normal blood pressure, with localized swelling and erythema.	B
Rapid drop in blood pressure upon exposure to an allergen, with angioedema.	D
Elevated blood pressure due to anxiety, with mild facial edema.	B

These 5 steps have been completed in less than 10 minutes for each model. The whole process within the AI environment can be displayed by accessing the following public pages of the human-AI conversations: Gierl et al’s [3] item [34] and TUS item [35].

### Step 6: Final Review by the Expert(s)

During this phase, the expert carries out a final review of the item model and cognitive model provided by AI, preferably with other experts.

In the previous steps, in order to keep the demonstration simple and due to the fact that there was not a significant inaccuracy in the AI-generated content, the expert did not demand any additional changes during the process in AI environment. But in the hybrid method, the 5 steps within the AI environment should be actively monitored by the expert, and if necessary, the expert should input prompts to make changes because AI is always prone to provide inaccurate content and deviate from providing a consistent template. Expert oversight, and involvement if necessary, is a strong necessity for creating high-quality item models and cognitive models.

Following the first 5 steps, which can be completed in less than 10 minutes, the expert should carry out one more round of review for the item model and the cognitive model generated through human-AI collaboration. It would be better if the expert conducts this review together with other experts to make sure that there is no inaccuracy, inconsistency, or inappropriate way of presentation. The expert should keep in mind that content generated by AI, in this case ChatGPT, is generated through a large language model, so it could “hallucinate” [36] some inaccurate information and relationships that are harmful for the output quality. Apart from that, in this step, the expert may prefer to add more elements and variables, such as age and gender, in a way that does not change the correct answers, in order for increasing the number of the items.

### Step 7: Item Generation Using a Non-AI Software

Finally, the expert inputs the final version of the item template and the constraints to a traditional template-based AIG tool (software without AI involvement), and then the software algorithmically produces multiple-choice items based on the input provided by the expert. It is crucial to emphasize that the expert must input the content accurately, as traditional software cannot handle inconsistent type of inputs, unlike AI in the previous steps. There is no difference between the traditional template-based method (stage 3) [10] and our hybrid method (step 7) in this regard. As is in the template-based method [10], Hybrid AIG also allows the software to generate hundreds of consistent items based on a single item model.

## Discussion

In this study, we used AI to generate item models and cognitive models for generating multiple-choice items by using these models for template-based AIG. We demonstrated that AI is capable of providing AIG templates for this purpose under the control of human expert. Leveraging AI in template development has significantly reduced the time investment from hours [10] to less than 10 minutes, and provided a smoother experience for experts in this challenging task [3].

In our hybrid AIG method, cognitive work required to be carried out by experts in the past [10] is now shared with AI. It switches the role of experts from “the creators of item-cognitive models from scratch” to “the reviewer of AI-generated content,” which brings an important efficiency to AIG without sacrificing consistency and accuracy. Our hybrid AIG method transcends the traditional template-based approach by marrying the “art” that comes from AI as a “black box” [25] with the “science” of algorithmic generation [10] under the oversight of expert as a “marriage registrar.” Practically, this balanced fusion under human guidance reduces the extraneous cognitive load [16] on experts by allocating the burdensome tasks to AI in order for enhancing human efficiency and allowing them to concentrate on refining and validating the AI-generated content.

Similar to our approach, a recent study successfully incorporated a large language model into the process of developing reading comprehension items [37]. While addressing a critical issue in item development for a non–health care setting, its direct application to medical education is challenging due to the inherent complexities of health professions education. Furthermore, this approach integrates AI only into generating unique sentences based on rules imposed by experts, leaving the essential cognitive work dependent on expert input, which remains inefficient for medical education. In our hybrid method, we use AI not only for generating unique sentences but also for development of item models and cognitive models as a whole, hence transforming the role of experts from the main “cognitive workers” to reviewers. This shift reduces cognitive effort for experts while maintaining their essential contribution for accurate and consistent items. Considering the importance of clarity and constraints in the templates, we still can suggest that it is possible and desirable to create specifications and instructions using artificial intelligence. Our research demonstrated that even a minimal human oversight can be sufficient for using AI in the creation of specifications and instructions, particularly in challenging domains such as medical education, which suggests even greater possibility for less complex tasks like reading comprehension. By dismissing the potential of AI in this regard by labeling it as “impossible,” humans might inadvertently limit AI’s capacity to enhance efficiency in cognitive work needed to be done. Thus, we propose leveraging AI more effectively rather than relegating it to a lesser role.

While noting the improved efficiency of the proposed hybrid for cognitive tasks, we emphasize the importance of rigorous human oversight, and consequently, accountability for automatically generated content. As demonstrated in a study by Zack et al [38], even a state of the art large language model can still be prone to perpetuate racial and gender bias. Adding to the complexity, such bias may not be visible at the level of a single question item or template, but as an effect of prolonged use of generative AI tools. The role of human examiners is to be aware of such risks and to implement bias mitigation strategies at different steps and levels of the AI process to prevent the injection of harmful stereotypes into the assessment of students’ skills.

Our study has some limitations. Although the templates generated by AI showed promising results, replicability depends on the consistency of the AI model, which is GPT-4 in this case. In addition, other AI models such as GPT-4o (Open AI), Claude (Anthropic), Gemini (Google), Llama (Meta), and Command *R*+ (Cohere) could lead to different outputs. While our study demonstrated that a hybrid AIG is possible, future research should explore this further by using different parent items across various settings to generate MCQs. As this is a proof-of-concept study, there is a lack of empirical evidence supporting the efficacy of the proposed hybrid AIG method, no qualitative reviews to assess the generated items’ quality, and a lack of quantitative item analysis since the items were not tested on medical students. However, it is still valuable because it has shown for the first time that generating plausible, and possibly useful, item templates using AI is possible in medical education. A recent study has provided empirical evidence, demonstrating that experts correctly identified the answers in MCQs generated by using hybrid AIG [39]. In the future studies, we are planning to generate more items using these templates and investigate their effectiveness using qualitative and quantitative methods. Moreover, a direct comparison of traditional template-based AIG and hybrid AIG could provide valuable evidence for effectiveness and efficiency. Another limitation is that we generated simple templates. There are multilayered templates for AIG [3], which require relatively complex structures, that might require from us to use different custom AIs for this purpose.

In conclusion, the hybrid AIG is a promising novel method that leverages AI in development of templates for template-based AIG that transforms the traditional role of experts from creators to reviewers. This shift can significantly reduce the cognitive burden on experts and streamline the item generation process while ensuring high-quality outcomes. We recommend piloting and improving the hybrid AIG in high-demand settings of increasing importance to investigate and improve its efficiency and quality benefits.

## Supplementary material

10.2196/65726 Multimedia Appendix 1 The prompt that has been used in the custom GPT.

## Abbreviations

AI: artificial intelligence
AIG: automatic item generation
MCQ: multiple-choice question

## References

[1] Pugh D, De Champlain A, Touchie C (2019). Plus ça change, plus c’est pareil: Making a continued case for the use of MCQs in medical education. Med Teach.

[2] Wrigley W, van der Vleuten CPM, Freeman A, Muijtjens A (2012). A systemic framework for the progress test: strengths, constraints and issues: AMEE Guide No. 71. Med Teach.

[3] Gierl MJ, Lai H, Tanygin V (2021). Advanced Methods in Automatic Item Generation.

[4] Kiyak YS, Budakoğlu Iİ, Coşkun Ö, Koyun E (2023). The first automatic item generation in Turkish for assessment of clinical reasoning in medical education. Tıp Eğitimi Dünyası.

[5] Kiyak YS, Coşkun Ö, Budakoğlu Iİ, Uluoğlu C (2023). Psychometric analysis of the first Turkish multiple-choice questions generated using automatic item generation method in medical education. Tıp Eğitimi Dünyası.

[6] Kıyak YS, Kononowicz AA, Górski S (2024). Multilingual template-based automatic item generation for medical education supported by generative artificial intelligence models ChatGPT and Claude. Bio-Algorithms Med-Syst.

[7] Leslie T, Gierl MJ (2023). Using automatic item generation to create multiple-choice questions for pharmacy assessment. Am J Pharm Educ.

[8] Lai H, Gierl MJ, Byrne BE, Spielman AI, Waldschmidt DM (2016). Three modeling applications to promote automatic item generation for examinations in dentistry. J Dent Educ.

[9] Falcão F, Costa P, Pêgo JM (2022). Feasibility assurance: a review of automatic item generation in medical assessment. Adv in Health Sci Educ.

[10] Gierl MJ, Lai H, Turner SR (2012). Using automatic item generation to create multiple-choice test items. Med Educ.

[11] Kosh AE, Simpson MA, Bickel L, Kellogg M, Sanford‐Moore E (2019). A cost–benefit analysis of automatic item generation. Educational Measurement.

[12] Pugh D, De Champlain A, Gierl M, Lai H, Touchie C (2016). Using cognitive models to develop quality multiple-choice questions. Med Teach.

[13] Gierl MJ, Lai H, Pugh D, Touchie C, Boulais AP, De Champlain A (2016). Evaluating the psychometric characteristics of generated multiple-choice test items. Applied Measurement in Education.

[14] Pugh D, De Champlain A, Gierl M, Lai H, Touchie C (2020). Can automated item generation be used to develop high quality MCQs that assess application of knowledge?. RPTEL.

[15] Gierl MJ, Lai H (2013). Evaluating the quality of medical multiple-choice items created with automated processes. Med Educ.

[16] van Merriënboer JJG, Sweller J (2010). Cognitive load theory in health professional education: design principles and strategies. Med Educ.

[17] Kıyak YS (2023). A ChatGPT prompt for writing case-based multiple-choice questions. Rev Esp Edu Med.

[18] Zuckerman M, Flood R, Tan RJB (2023). ChatGPT for assessment writing. Med Teach.

[19] Laupichler MC, Rother JF, Grunwald Kadow IC, Ahmadi S, Raupach T (2024). Large language models in medical education: comparing ChatGPT- to human-generated exam questions. Acad Med.

[20] Kıyak YS, Kononowicz AA (2024). Case-based MCQ generator: A custom ChatGPT based on published prompts in the literature for automatic item generation. Med Teach.

[21] Kıyak YS, Coşkun Ö, Budakoğlu Iİ, Uluoğlu C (2024). ChatGPT for generating multiple-choice questions: Evidence on the use of artificial intelligence in automatic item generation for a rational pharmacotherapy exam. Eur J Clin Pharmacol.

[22] Indran IR, Paranthaman P, Gupta N, Mustafa N (2024). Twelve tips to leverage AI for efficient and effective medical question generation: A guide for educators using Chat GPT. Med Teach.

[23] Ngo A, Gupta S, Perrine O, Reddy R, Ershadi S, Remick D (2024). ChatGPT 3.5 fails to write appropriate multiple choice practice exam questions. Acad Pathol.

[24] Lee H (2024). The rise of ChatGPT: exploring its potential in medical education. Anatomical Sciences Ed.

[25] von Eschenbach WJ (2021). Transparency and the black box problem: why we do not trust AI. Philos Technol.

[26] Bulut O, Beiting-Parrish M, Casabianca JM (2024). The rise of artificial intelligence in educational measurement: opportunities and ethical challenges. arXiv.

[27] Han Z, Battaglia F, Udaiyar A, Fooks A, Terlecky SR (2024). An explorative assessment of ChatGPT as an aid in medical education: Use it with caution. Med Teach.

[28] Appelbaum SH (1997). Socio‐technical systems theory: an intervention strategy for organizational development. Manag Decis.

[29] Mesko B (2023). The ChatGPT (generative artificial intelligence) revolution has made artificial intelligence approachable for medical professionals. J Med Internet Res.

[30] White J, Fu Q, Hays S (2023). A prompt pattern catalog to enhance prompt engineering with chatgpt. arXiv.

[31] Kıyak YS (2024). Beginner-level tips for medical educators: guidance on selection, prompt engineering, and the use of artificial intelligence chatbots. Med Sci Educ.

[32] Masters K, Benjamin J, Agrawal A, MacNeill H, Pillow MT, Mehta N (2024). Twelve tips on creating and using custom GPTs to enhance health professions education. Med Teach.

[33] Item Model Maker for AIG. ChatGPT.

[34] Item model 1. ChatGPT.

[35] Item model 2. ChatGPT.

[36] Masters K (2023). Medical Teacher ’s first ChatGPT’s referencing hallucinations: lessons for editors, reviewers, and teachers. Med Teach.

[37] Sayin A, Gierl M (2024). Using OpenAI GPT to generate reading comprehension items. Educational Measurement.

[38] Zack T, Lehman E, Suzgun M (2024). Assessing the potential of GPT-4 to perpetuate racial and gender biases in health care: a model evaluation study. Lancet Digit Health.

[39] Kıyak YS, Emekli E, Coşkun Ö, Budakoğlu Iİ (2025). Keeping humans in the loop efficiently by generating question templates instead of questions using AI: Validity evidence on Hybrid AIG. Med Teach.

